# Gradually focused fine-grained sketch-based image retrieval

**DOI:** 10.1371/journal.pone.0217168

**Published:** 2019-05-28

**Authors:** Ming Zhu, Chun Chen, Nian Wang, Jun Tang, Wenxia Bao

**Affiliations:** School of Electronics and Information Engineering, Anhui University, Hefei, China; Beijing University of Technology, CHINA

## Abstract

This paper focuses on fine-grained image retrieval based on sketches. Sketches capture detailed information, but their highly abstract nature makes visual comparisons with images more difficult. In spite of the fact that the existing models take into account the fine-grained details, they can not accurately highlight the distinctive local features and ignore the correlation between features. To solve this problem, we design a gradually focused bilinear attention model to extract detailed information more effectively. Specifically, the attention model is to accurately focus on representative local positions, and then use the weighted bilinear coding to find more discriminative feature representations. Finally, the global triplet loss function is used to avoid oversampling or undersampling. The experimental results show that the proposed method outperforms the state-of-the-art sketch-based image retrieval methods.

## Introduction

### Motivation

In recent years, with the emergence and development of touch screen devices and convolution neural networks[1] (CNNs), fine-grained sketch-based image retrieval (FG-SBIR) has been widely used. Sketch-based image retrieval[2–9] is an important direction of content-based image retrieval, but compared with some content-based image retrieval[10–13] that requires sample queries, sketch-based image retrieval can get rid of this shackle. You can get more intuitive and accurate information by just drawing a few strokes based on the impression of the object[14]. Furthermore, FG-SBIR has clearly more commercially valuable than traditional sketch-based image retrieval focus on category-level. For example, given a sketch of shoes, we would like to search for the specific shoes corresponding to this sketch, instead of just searching for shoes.

FG-SBIR is a very challenging problem: (1) Free-hand sketches contain only simple contour information, but the retrieved images are often rich in color and texture information, which belongs to cross-domain retrieval problem. (2) Free-hand sketches are highly abstract, and may be highly misplaced with the image to be retrieved. It is difficult to match the details of the sketch with the image. (3) Different people have different painting habits. Even the same image may be drawn in different styles, especially in the subtleties, and many candidate images have only subtle differences, which leads to difficulties in matching.

**Fig 1:**
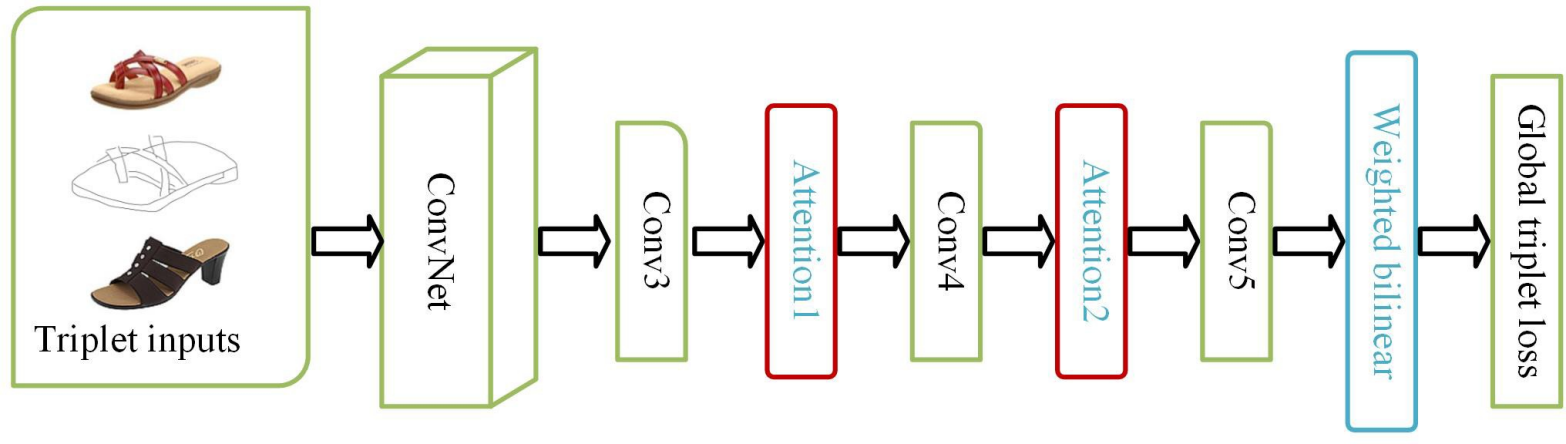
Model framework.

There are many existing works on FG-SBIR[15–18]. [15–17] all adopt CNNs developed in recent years. Specifically, [15] uses the traditional triplet network, which uses the output of the full connection (FC) layers to align the domain. However, it does not take into account that the high level of the network will lose details and the triplet loss function requires a good strategy for triplet selection, so it is easy to fall into under-fitting or over-fitting. [16] adds an attention model to highlight the details and uses a shortcut structure to connect coarse-grained information with fine-grained information after global average pooling (GAP). However, simply adding the attention model to the last layer of the convolutional layer can not accurately capture the details, while the GAP can only obtain the first-order statistics, ignoring the correlation between features. In view of the above problems, this paper designs a gradually focused bilinear attention model. First, because the higher the network level, the more abstract the information captured, an object may have multiple details, adding the attention model to different convolutional layers not only improves the accuracy of focusing details, but also pays attention to different parts at the same time. Then, the attention model and the weighted bilinear coding are combined to obtain more discriminant feature representation. Finally, the global loss function is used to avoid oversampling or undersampling. The model structure is shown in Fig 1.

All in all, the contributions of this paper are summarized as follows:

1. A novel gradually focused bilinear attention model is proposed. In particular, the attention model can effectively focus on multiple details.
2. A weighted bilinear coding is introduced to aggregate features to obtain more useful second-order statistical information.
3. A global loss function is introduced to accelerate convergence, reduce the impact of sampling strategy, and avoid oversampling or undersampling.

### Related work

There are many related research works in image retrieval, such as cross-modal search based on hetero-manifold regularisation (HMR)[19], person re-identification based on cross-view binary identities (CBI)[20] and so on, are all related to image retrieval and have achieved good results. This paper focuses on FG-SBIR, and we will briefly review the related work about FG-SBIR in this section.

The use of free-hand sketches for retrieval only requires the user to have a mental image of the corresponding object, so it has great research value and commercial value, and has been widely concerned. However, one of the biggest obstacles is the lack of datasets. In 2012, the first large-scale drawing dataset TU-Berlin[21] appeared. It contains 20000 sketches, a total of 250 categories, each contains 80 sketches, mainly used for sketch recognition. But since these sketches represent only a certain class of concepts without photographs and detailed classification, they are not suitable for fine-grained matching. And before 2015, feature extraction still relies on manual, it is difficult to extract some details, so the research progress of fine-grained direction is not outstanding.

With the great achievements of CNN in the field of image, many researchers began to apply CNN to sketch recognition. However, because of the lack of the normal images’ color and texture information, the model at that time did not achieve significant results in sketch recognition. In 2015, YU et al. proposed ‘sketch-a-net’[22] for sketch recognition problem, which achieved good results by using larger filter size and pool size because the sketches lack texture information, but limited to category-level recognition. Later, YU et al. established three datasets[15,16] for fine-grained image retrieval: QMUL-Shoe, QMUL-Chair, handbag, and designed a special training method. Specifically, sketch-a-net was trained from scratch with the edge graph extracted from the dataset ImageNet-1k and was fine-tuned using the sketch in TU-Berlin to realize category-level image recognition. Then the sketch-photos pairs selected from ImageNet and TU-Berlin[21] were pre-trained through the triplet network, each branch of the triplet network was a trained sketch-a-net, and then three datasets are used to fine-tune the pre-trained triplet network to achieve fine-grained image retrieval.

The triplet network is also used in this paper, as shown in Fig 1. [23,24] shows that after many pool and FC layers, spatial fine-grained details have disappeared and cannot be recovered, so we abandon the FC layer and only adopt the convolution layer.

Because the datasets are relatively small, and easy to produce over-fitting, so a simple clipping method is used to do data augmentation. A spatial-semantic attention model in [16] is used to focus on key points, but the authors point out that the focus is occasionally in the wrong place, so this paper uses a gradual focusing approach to highlight the details of the sketches and reduce the possibility of errors. In order to improve efficiency and practicability, most papers based on CNNs use GAP to aggregate features, but due to the lack of feature correlation, GAP often leads to sub-optimization[25]. Therefore, the weighted bilinear coding model[25] is used to obtain more distinguishing features. In order to improve the ranking results, there are many measurement methods, such as Euclidean distance [26], Hamming distance [19,20] and so on[27]. The measurement methods and loss function commonly used in triplet networks is the first order distance function[28] and hinge loss. The loss function only considers the relationship between each triplet and ignores the global information. The quality of triplet network depends largely on the strategy of triplets selection. If the difference between the anchor samples and the positive samples are mostly smaller than that between the anchor samples and the negative samples, that is to say, the triplets are too simple, which will lead to slow convergence. On the contrary, if most training situations are very difficult, it will lead to over-fitting[29]. In order to overcome this defect, we add the global triplet loss function according to the method of [30].

The rest of this paper is organized as follows. The proposed FG-SBIR method is detailed in Section 2. In Section 3, we report and analyze the experimental results on three datasets. The conclusions are given in Section 4.

## Method

As shown in Fig 1, the FG-SBIR problem is solved as a ranking problem. Suppose that given a photo set *P* = {*p*_1_,*p*_2_,⋯,*p*_*N*_} and a sketch set *S* = {*s*_1_,*s*_2_,⋯,*s*_*N*_}, there are *N* photos and *N* sketches respectively, in which each photo corresponds to a sketch drawn by it. We obtain triplets ${\left\{\left(s_{i},p_{i}^{+},p_{i}^{-}\right)\right\}}_{i=1}^{N}$ via the method in [15,16], where *s* denotes sketches, *p*^*+*^ and *p*^*-*^ represent positive and negative samples, and those samples are edge maps extracted from datasets. Our goal is to reduce the distance between the sketch and the positive sample, and to widen the distance between the sketch and the negative sample. When testing, give a query sketch *s* and *M* candidate photos, we extract the edge maps of the candidate photos and calculate the distance between the query sketch and the edge maps of the candidate photos. The smaller the distance, the higher the score is, which means that the greater the possibility of correctly matching images:

$$Rs\left(B\left(s\right),B\left(p\right)\right)=-D\left(B\left(s\right),B\left(p\right)\right)=-{\left\|B\left(s\right)-B\left(p\right)\right\|}_{2}^{2}$$ (1)

where *B*(⋅) denotes the feature map that has been learned.

### Gradually focused attention model

For the FG-SBIR problem, one of the most challenging problems is how to extract the most representative local features. When we look at a image, our attention is always attracted to one particular part, and less to others. This means that our attention distribution on a image is different. Based on this mechanism of the human brain, the attention model in deep learning is developed. Popularly speaking, attention models are weighted changes in target data to highlight the important position.

**Fig 2:**
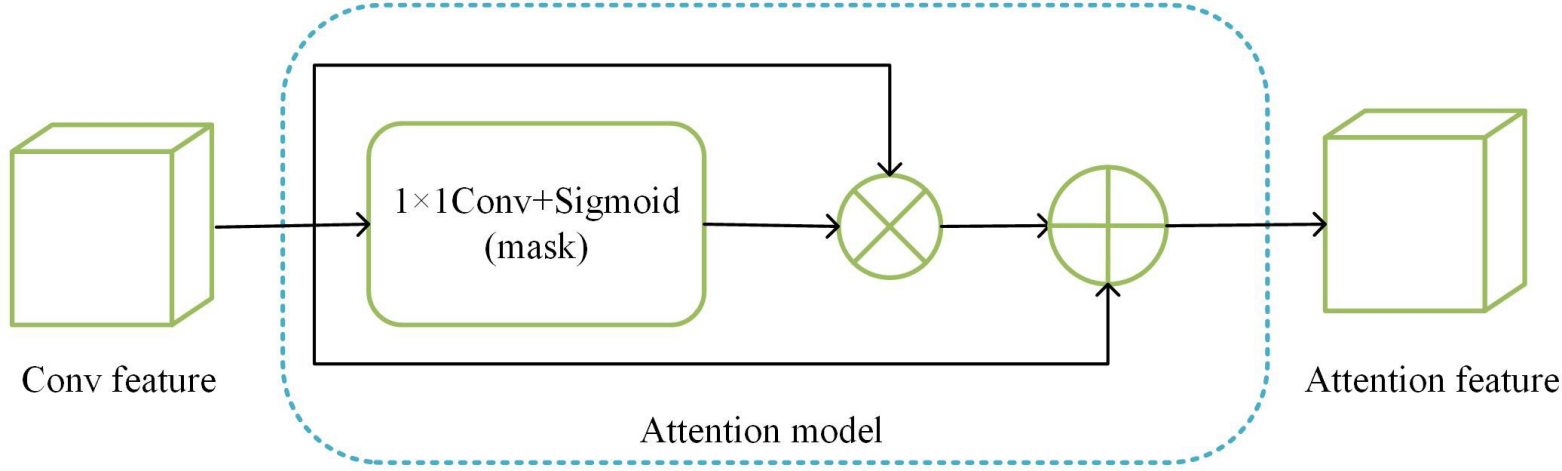
Attention model.

The attention model used in this paper is shown in Fig 2. It consists of a 1×1 convolution layer and a sigmoid layer. Sigmoid layer can pay attention to different possibly unique locations. Assuming that the input is a three-dimensional convolution feature map as $F\in {ℜ}^{H\times W\times C}$, where *H* and *W* are the feature map size and *C* is the number of feature channels, we can calculate the two-dimensional mask $M_{l}\in {ℜ}^{H\times W},l\in \left\{1,2\right\}$ as:

$$M_{l}=\psi_{mask_{l}}\left(F\right)$$ (2)

where *M*_*l*_ represents the mask corresponding to the *l-th* attention model. $\psi_{mask_{l}}\left(\cdot \right)$ represents the *l-th* mask generator. Experiments show that the sigmoid function can distinguish the details more clearly.

The value of each location of the mask represents the importance of the input feature map corresponding to the spatial position, and multiplies the input feature map with the mask to obtain the weighted feature map such as:

$$F_{M_{l}}=F⊗M_{l}$$ (3)

Where ‘⊗’ is element-wise product so that we can highlight the key locations learned, but since there may be some errors in the positions learned, some useful information may be lost if $F_{M_{l}}$ is directly input to the next layer. To reduce this error, we add the input feature map to the weighted feature map according to the method of [16]. Output such as:

$$F_{att\_l}=F+F_{M_{l}}=F+F⊗M_{l}$$ (4)

where ‘+’ is element-wise sum. This accentuates representative parts and preserves other potentially useful information. We use *F*_*att_l*_ as the input to the next layer of convolution. Although we expect the attention model to highlight distinctive local locations, however, due to the influence of some noise and spatial misaligned, adding attention model only in the last layer of CNN often notices the wrong position. Therefore, this paper adds attention model to the multiple convolution layer to focus on the discriminative feature.

### Weighted bilinear coding

**Fig 3:**
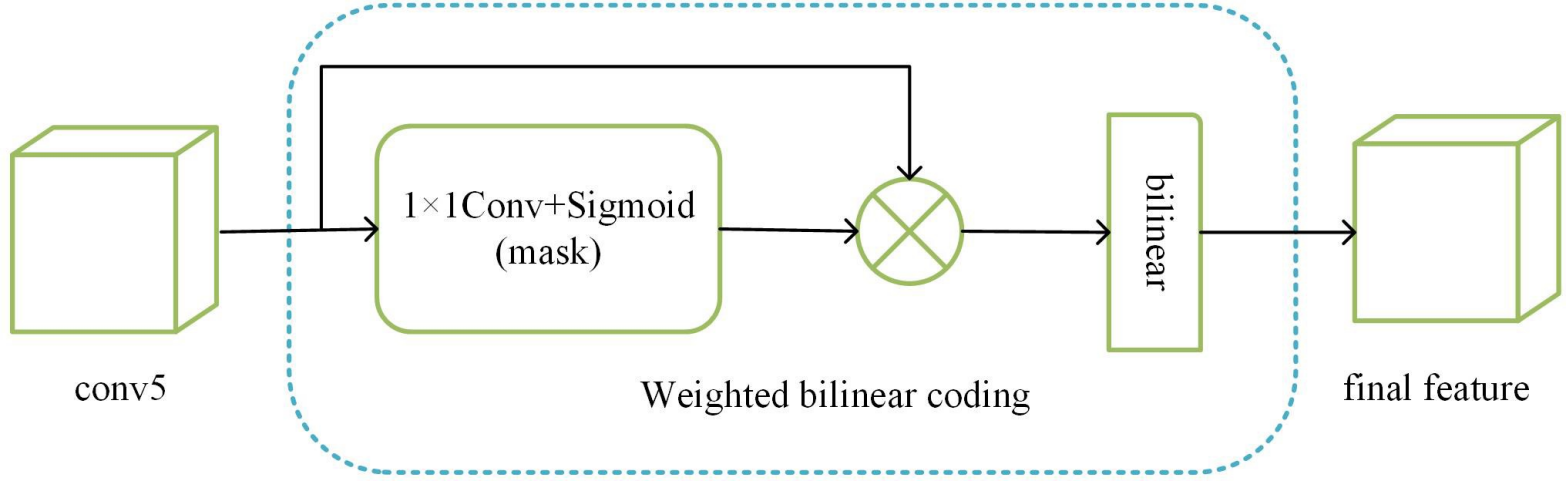
Weighted bilinear coding model.

As we abandon the FC layer, if we want to send the feature map of conv5 into the loss layer, we need to reshape the feature map to a vector. Most of the existing papers use GAP, but it can only capture first-order statistics and ignore the interaction between each element of the feature map. The bilinear model proposed in [31] can improve the performance of multiple visual tasks by collecting second-order information in the form of translation invariants. Our weighted bilinear coding model is shown in Fig 3.

Assuming that the input feature map of conv5 is $F_{conv5}\in {ℜ}^{H\times W\times C}$, after bilinear coding, we can get the following feature of the output:

$$B=\sum_{i=1}^{H}\sum_{j=1}^{W}F_{conv5}{\left(i,j\right)}^{T}F_{conv5}\left(i,j\right)$$ (5)

where $F_{conv5}\left(i,j\right)\in {ℜ}^{1\times C}$ is the local feature at the (i,j)-th location. Although bilinear coding can get richer information than GAP, but [25] points out that this approach considers each location equally important, but in practice, the effects of different local features are different. So we take a similar approach to [25], by learning a mask to distinguish the importance of different locations, and the learning mask was similar to the previous attention model, and consisting of a 1×1 convolution layer and a sigmoid layer. But since we need a feature that can highlight the key points, we only multiply the feature map of conv5 with the mask, as shown in Fig 3. We calculate the weighted bilinear coding method as follows:

$$B=\sum_{i=1}^{H}\sum_{j=1}^{W}{\left(M\left(i,j\right)F_{conv5}\left(i,j\right)\right)}^{T}\left(M\left(i,j\right)F_{conv5}\left(i,j\right)\right)$$ (6)

where *M*(*i*,*j*) represents the mask learned. According to formula (6), a C×C feature map is obtained, and we reshape it into a C^2^ length feature vector as the feature representation. And finally, we normalize it before we send it to the loss function.

### Global triplet loss

In triplet networks, the traditional triplet loss function is often used. For a given triplet $\left(s_{i},p_{i}^{+},p_{i}^{-}\right)$, the loss function can be written as:

$$L=\max\left(0,\;D\left(B\left(s\right),B\left(p^{+}\right)\right)-D\left(B\left(s\right),B\left(p^{-}\right)\right)+t\right)$$ (7)

where *D*(⋅) denotes euclidean distance, *B*(⋅) denotes the feature map of the corresponding network’ output, t is the required margin. The main idea of triplet loss function is to minimize the mean value of the distance between the same class and to maximize the mean value of the distance between different classes. Although triplet loss function is widely used, it needs a certain sampling strategy. It is difficult to converge when the triplet is too simple (i.e., formula (7) is easy to satisfy), and it is easy to over-fit when the triplet is too difficult (i.e., formula (7) is difficult to satisfy). In order to solve the problem of under-sampling and over-sampling, According to the idea of [30], we add global loss to the traditional triplet loss function as:

$$L=\left(\sigma^{2+}+\sigma^{2-}\right)+\lambda \max\left(0,\;\mu^{{}_{+}}-\mu^{‐}+t\right)$$ (8)

where $\mu^{+}=\sum_{i=1}^{N}D\left(B_{i}\left(s\right),B_{i}\left(p^{+}\right)\right)/N$, $\sigma^{2+}={\sum_{i=1}^{N}\left(D\left(B_{i}\left(s\right),B_{i}\left(p^{+}\right)\right)-\mu^{+}\right)}^{2}/N$, $\mu^{-}=\sum_{i=1}^{N}D\left(B_{i}\left(s\right),B_{i}\left(p^{-}\right)\right)/N$, $\sigma^{2-}={\sum_{i=1}^{N}\left(D\left(B_{i}\left(s\right),B_{i}\left(p^{-}\right)\right)-\mu^{-}\right)}^{2}/N$, *μ*^+^ and *σ*^2+^ denotes the mean and variance of the distance between the sketch and the positive sample in the same batch respectively. *μ*^−^ and *σ*^2−^ denotes the mean and variance of the distance between the sketch and the negative sample in the same batch respectively. The purpose of this loss function is to minimize the variance between the same class and different classes, maximize the mean values of different classes, and minimize the mean values of the same class.

### Experimental procedure

The basic network framework used in this paper is triplet network, in which each layer of network information is shown in Table 1.

**Table 1:** The architecture of CNN.

Layer	Type	Filter Size	Filter Num	Stride	Pad	Output Size
	Input	-	-	-	-	225×225
**L1**	Conv	15×15	64	3	0	71×71
	Relu	-	-	-	-	71×71
	Maxpool	3×3	-	2	0	35×35
**L2**	Conv	5×5	128	1	0	31×31
	Relu	-	-	-	-	31×31
	Maxpool	3×3	-	2	0	15×15
**L3**	Conv	3×3	256	1	1	15×15
	Relu	-	-	-	-	15×15
**Attention L1**	Conv	1×1	1	1	0	15×15
	Sigmoid	-	-	-	-	15×15
**L4**	Conv	3×3	256	1	1	15×15
	Relu	-	-	-	-	15×15
**Attention L2**	Conv	5×5	128	1	0	31×31
	Relu	-	-	-	-	31×31
**L5**	Conv	3×3	256	1	1	15×15
	Relu	-	-	-	-	15×15
	Maxpool	3×3	-	2	0	7×7
**Weighted bilinear Layer**	Conv	1×1	1	1	0	7×7
	Sigmoid	-	-	-	-	7×7
	Bilinear pool	-	-	-	-	1×65536

The overall training procedure is summarized in Algorithm 1.

Algorithm 1            Training procedure

Input: Set of triplets ${\left\{\left(s_{i},p_{i}^{+},p_{i}^{-}\right)\right\}}_{i=1}^{N}$; Total epochs *T* of deep optimization.

Output: Weights of each convolutional layers.

1: Use [15] pre-trained network weights as the initialization weights

2: For *t* = 1, …,*T* epoch do

3: Get the feature map of conv3, and calculate the attended feature map by formula (2,3,4), then input it to the conv4.

4: Repeat the operation of step 3 for the output of the conv4, and input to the conv5.

5: The feature map of the conv5 is aggregated by the formula (6) and calculated the loss value by the formula (8).

6: Update the deep parameters with the gradient descent method

7: End

## Experiment

### Datasets and experimental details

**Fig 4:**
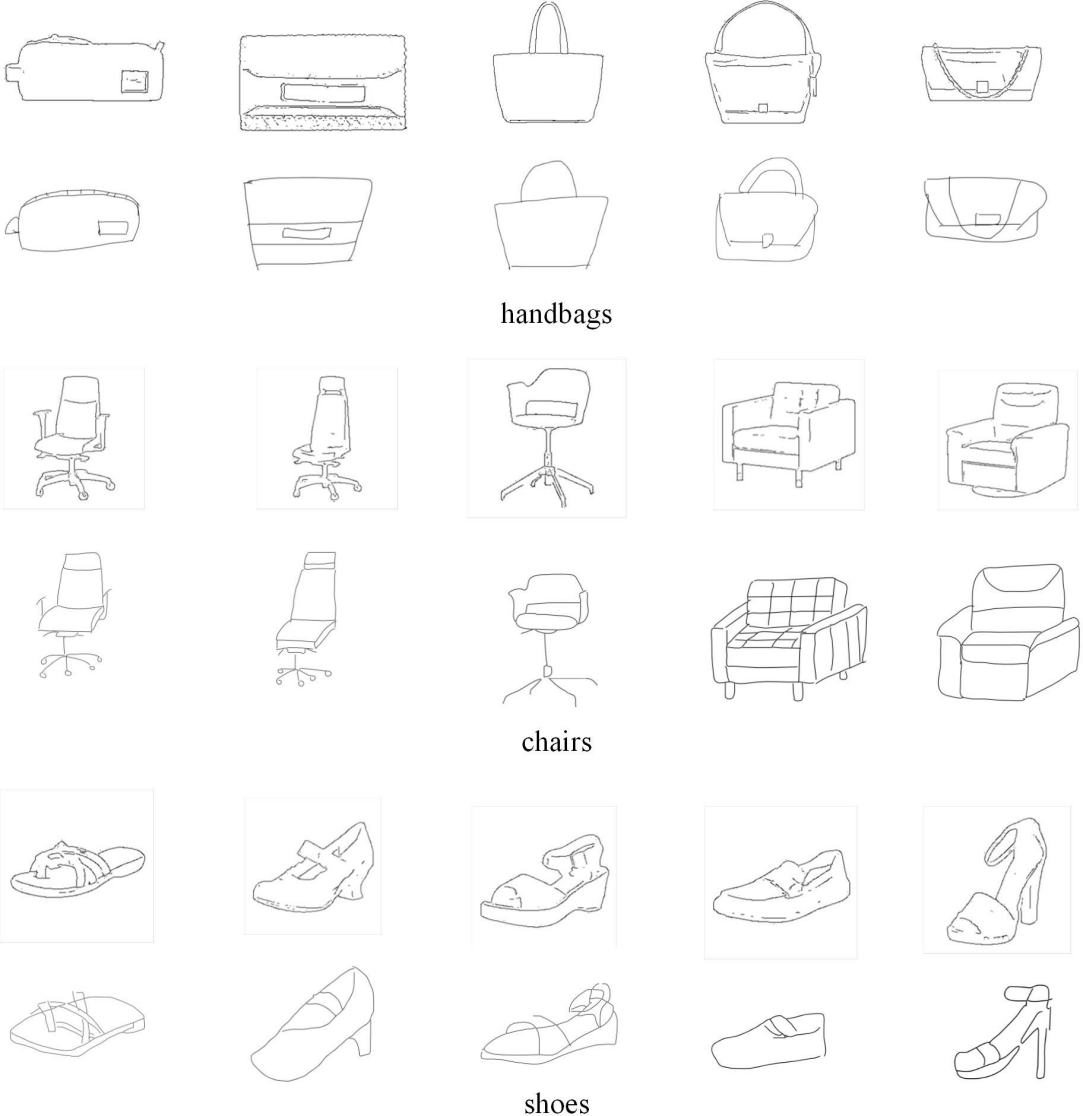
Image examples of the three datasets.

This paper has conducted experiments on three datasets. Some image examples of the three datasets are shown in Fig 4. QMUL-Shoe[15] contains a total of 419 shoe sketch-photo pairs and edge images extracted from the photos, of which 304 pairs are used for training and 105 pairs for testing. QMUL-Chair[15] contains a total of 297 chair sketch-photo pairs and edge images extracted from photos, of which 200 pairs are used for training and 97 pairs for testing. The two datasets have 13680 and 9, 000 manually annotated triplets respectively. Handbag[16] contains a total of 568 handbags sketch-photo pairs and edge images extracted from photos, of which 400 pairs are used for training and 168 pairs for testing. Unlike the first two datasets, it does not have a manually annotated triplet, but instead takes a photo matching the sketch as a positive sample and randomly selects one from the remaining photos in the training set as a negative sample. These photos were collected from online shopping sites and sketches were drawn by hand. The positive and negative samples we use in training and testing are edge images extracted from these photos.

We get the weight of pre-training according to the method of [15]. In training, because the three training sets are small, we use the method of clipping to augment the data. The image size in the dataset is 256×256. In the actual input, we use the four corners of the original image as the vertices to crop out four 225×225 images. In addition, with the center cropping and flipping them, the final 256×256 original image can get ten 225 × 225 images. In practice, these augmented images are trained in the same batch. A total of 320 triplets were trained at one time.

### Comparative results

We chose two baseline experiments to compare: Triplet SN[15] and DSSA[16]. Triplet SN is the first end-to-end depth model for FG-SBIR problem. It uses a clever method to pre-train and fine-tune with the traditional triplet network, in which the basic network architecture is sketch-a-net. DSSA adds attention model to Triplet SN and proposes a higher-order distance function as a loss function. In this paper, the attention model is modified on the basis of DSSA, and the weighted bilinear coding is used to obtain more useful feature information. In addition, the loss function with global constraints can effectively avoid the problems caused by over-sampling and under-sampling. The experimental results are shown in Tables 2–4.

**Table 2:** Comparison with baseline on QMUL-Shoe.

QMUL-Shoe	Acc.@1	Acc.@10
**Triplet SN**	52.17%	92.17%
**DSSA**	61.74%	94.78%
**Our model**	65.22%	95.65%

**Table 3:** Comparison with baseline on QMUL-Chair.

QMUL-Chair	Acc.@1	Acc.@10
**Triplet SN**	72.16%	98.96%
**DSSA**	81.44%	95.88%
**Our model**	87.63%	97.94%

**Table 4:** Comparison with baseline on Handbag.

Handbag	Acc.@1	Acc.@10
**Triplet SN**	39.88%	82.14%
**DSSA**	49.40%	82.74%
**Our model**	57.74%	90.48%

We use top-1 and top-10 as the criteria for detecting the correct rate, that is, in the final ranking, the probability of the highest score being the correct match and the probability of correct matching in the top ten. From the result, we can see that our model is superior to other baselines.

### Ablation study

We introduce three novel components in our model: attention model(AM), weighted bilinear coding(WBC) and global triplet loss(GTL). In order to evaluate the contribution of each component, We compare our full model with models that lack one component and two components respectively. Tables 5–7 shows the performance of each component.

**Table 5:** Contributions of different component on QMUL-Shoe.

QMUL-Shoe	Acc.@1	Acc.@10
**without WBC and GTL**	58.26%	94.78%
**without AM and GTL**	58.26%	96.52%
**without AM and WBC**	61.74%	92.17%
**without AM**	64.35%	94.78%
**without WBC**	59.13%	94.78%
**without GTL**	60.87%	92.17%
**full model**	65.22%	95.65%

**Table 6:** Contributions of different component on QMUL-Chair.

QMUL-Chair	Acc.@1	Acc.@10
**without WBC and GTL**	84.41%	96.91%
**without AM and GTL**	86.60%	96.91%
**without AM and WBC**	82.47%	96.91%
**without AM**	82.47%	98.97%
**without WBC**	84.54%	96.91%
**without GTL**	85.57%	97.94%
**full model**	87.63%	97.94%

**Table 7:** Contributions of different component on Handbag.

Handbag	Acc.@1	Acc.@10
**without WBC and GTL**	52.38%	83.33%
**without AM and GTL**	54.17%	83.33%
**without AM and WBC**	55.36%	86.31%
**without AM**	37.50%	75.60%
**without WBC**	52.38%	86.31%
**without GTL**	51.79%	86.31%
**full model**	57.74%	90.48%

In the experiments without weighted bilinear coding and global triplet loss, we use GAP and traditional triplet loss function instead. We can see that the best results can be obtained only when these components are all present.

## Conclusion

We introduce a novel gradually focused attention model for FG-SBIR. The gradually focused attention model can capture many subtle representative local information, and the weighted bilinear coding can aggregate the convolution features more discriminatively and improve the representation ability. The global triplet loss function reduces the effect of over-sampling or under-sampling. By combining these three components, we demonstrate the effectiveness of this method on three datasets.
